# Coronavirus Disease (COVID)-19 and Diabetic Kidney Disease

**DOI:** 10.3390/ph14080751

**Published:** 2021-07-30

**Authors:** Swayam Prakash Srivastava, Rohit Srivastava, Subhash Chand, Julie E. Goodwin

**Affiliations:** 1 Department of Pediatrics, Yale University School of Medicine, New Haven, CT 06520, USA; 2 Vascular Biology and Therapeutics Program, Yale University School of Medicine, New Haven, CT 06511, USA; 3 Laboratory of Medical Transcriptomics, Department of Endocrinology, Nephrology Services, Hadassah Hebrew-University Medical Center, Jerusalem 91905, Israel; rohitsri.cdri@gmail.com; 4 Department of Anesthesiology, University of Nebraska Medical Center, Omaha, NE 68198, USA; subhash.ecc@gmail.com

**Keywords:** severe acute respiratory syndrome coronavirus 2 (SARS-CoV-2), coronavirus disease (COVID)-19, diabetes, kidney diseases, diabetic kidney disease, dipeptidyl peptidase (DPP)-4, AMP-activated protein kinase (AMPK), microRNAs, LncRNAs, circular RNAs, angiotensin-converting enzyme (ACE), ACE2, epithelial-to-mesenchymal transition, endothelial-to-mesenchymal transition, COVID-19 associated nephropathy

## Abstract

The present review describes COVID-19 severity in diabetes and diabetic kidney disease. We discuss the crucial effect of COVID-19-associated cytokine storm and linked injuries and associated severe mesenchymal activation in tubular epithelial cells, endothelial cells, and macrophages that influence neighboring cell homeostasis, resulting in severe proteinuria and organ fibrosis in diabetes. Altered microRNA expression disrupts cellular homeostasis and the renin-angiotensin-system, targets reno-protective signaling proteins, such as angiotensin-converting enzyme 2 (ACE2) and MAS1 receptor (MAS), and facilitates viral entry and replication in kidney cells. COVID-19-associated endotheliopathy that interacts with other cell types, such as neutrophils, platelets, and macrophages, is one factor that accelerates prethrombotic reactions and thrombus formation, resulting in organ failures in diabetes. Apart from targeting vital signaling through ACE2 and MAS, severe acute respiratory syndrome coronavirus 2 (SARS-CoV-2) infections are also associated with higher profibrotic dipeptidyl transferase-4 (DPP-4)-mediated mechanisms and suppression of AMP-activated protein kinase (AMPK) activation in kidney cells. Lowered DPP-4 levels and restoration of AMPK levels are organ-protective, suggesting a pathogenic role of DPP-4 and a protective role of AMPK in diabetic COVID-19 patients. In addition to standard care provided to COVID-19 patients, we urgently need novel drug therapies that support the stability and function of both organs and cell types in diabetes.

## 1. Introduction

Coronavirus disease 2019 (COVID-19) is caused by severe acute respiratory syndrome coronavirus 2 (SARS-CoV-2), a beta-coronavirus that belongs to the family Coronaviridae and order Nidovirales [1]. It is an enveloped, positive-stranded RNA virus with a genome of 27 to 32 kb, packed inside of a helical capsid of nucleocapsid protein (N). The envelope contains membrane (M), envelope (E), and spike proteins (S). The proteins M and E are involved in virus assembly, while the spike protein S mediates virus entry into host cells [1]. The spike is a critical determinant of the viral host range and a major inducer of host immune responses.

SARS-CoV-2 virus is transmitted from human to human and causes respiratory tract infection that can progress to severe lung infection and serious health complications [2,3,4]. It infects respiratory epithelial cells using Angiotensin-converting enzyme 2 (ACE2) and transmembrane protease, serine 2 (TMPRSS2) receptors [5]. The SARS-CoV-2 infection can be asymptomatic, mild, or life-threatening [4,6,7,8,9]. Regardless of the severity of the symptoms, an infected individual is more likely to spread the infection and poses a great risk to vulnerable populations, such as immunocompromised individuals and those with diabetes, hypertension, and asthma [10,11,12].

Diabetes mellitus (DM) is often recognized as an independent risk factor for developing respiratory tract infections [13]. From the onset of COVID-19, a relationship between the clinical course of severe acute respiratory syndrome (SARS) and blood glucose levels has been established. Patients with both diabetes and COVID-19 infection can be harder to treat, due to fluctuations in blood glucose levels, and possibly, the presence of other related complications. A compromised immune system makes it harder to fight the virus and leads to a more extended recovery period. In addition, hyperglycemia helps the virus thrive effectively, thus making it difficult to manage the infection in diabetic patients. Significant alterations in the immune system of diabetic patients are observed, such as significant alterations in humoral and cell-mediated immune function. These factors interfere with the immune and pulmonary function of diabetic patients with COVID-19, which further increases mortality risk.

A study by Li et al. in 199 patients conclude that 14.5% of patients who have both diabetes and COVID-19 pneumonia die, whereas 5.7% of nondiabetic COVID-19 patients die, suggesting that diabetes increases mortality in COVID-19 patients [13]. The antidiabetic drug treatment category was associated with decreasing odds of death. In this study, compared to nondiabetic patients, diabetic patients had lower lymphocyte levels and a lymphocyte count less than 0.6 × 10^9^/L at admission, which were associated with an increased odds of death. It is worth noting that lymphocytopenia is common in critically ill Middle East respiratory syndrome (MERS) patients [13], which commonly has been observed associated to microvascular injuries and thrombosis in COVID-19 patients [14]. In another study, Yang et al. reported that lymphocytopenia occurs in more than 80% of critically ill patients with COVID-19 [15], and is a prominent feature because SARS-CoV infection damages the cytoplasmic component of the lymphocyte and destroys the cell [13,16]. Hence, it is postulated that necrosis or apoptosis of lymphocytes also induces lymphocytopenia in critically ill patients with COVID-19. The severity of lymphocytopenia may reflect exacerbation of the disease [13].

Rodelo et al. reported that high D-dimer levels in patients with critical infection or sepsis at admission was associated with increased odds of death [17]. D-dimer levels were quite different between diabetic and nondiabetic groups [13]. D-dimer, an activation marker of fibrinolysis, is dramatically increased in COVID-19 patients with diabetes [13]. Elevation of D-dimer is a prognostic factor in patients with pneumonia and sepsis, and elevated levels of D-dimer were also found to be a risk factor for mortality in patients with COVID-19 [13,17]. Perhaps not surprisingly, microvascular injury and thrombosis are also associated with the severity of COVID-19 infection [14]. It will be interesting to study if anticoagulant administration impacts these conditions in patients with SARS-CoV-2 infection. In this review, we focus on the mechanisms by which COVID-19 influences diabetes and diabetic kidney disease (DKD).

## 2. Diabetic Kidney Disease

Kidney failure is associated with diabetes mellitus. Over time, hyperglycemia damages the glomeruli, leading to kidney failure. Around 20–30% of patients with diabetes develop diabetic nephropathy (DN). Regardless of insulin administration, diabetic patients are susceptible to nephropathy. Current treatments include angiotensin-converting enzyme inhibitors (ACEi), angiotensin II receptor blockers (ARBs), and statins; however, these drugs reduce proteinuria by hemodynamic perturbations and do not address the underlying factors that incite and perpetuate DN [18,19,20,21,22]. A number of renal protective molecules, such as SGLT-2 inhibitors, mineralocorticoid receptor antagonists, endothelin antagonists, glucagon-like peptide-1 receptor agonists (GLP1-RA), DPP-4 inhibitors, and statins, have been studied in preclinical models and randomized controlled trials (RCTs) and significant advancement in recent years have been achieved [23,24,25,26,27,28,29,30,31,32,33,34,35,36]. However, the complicated pathogenesis of DN contributes to the sub-optimal treatment options available [19,20,37].

**Figure 1:**
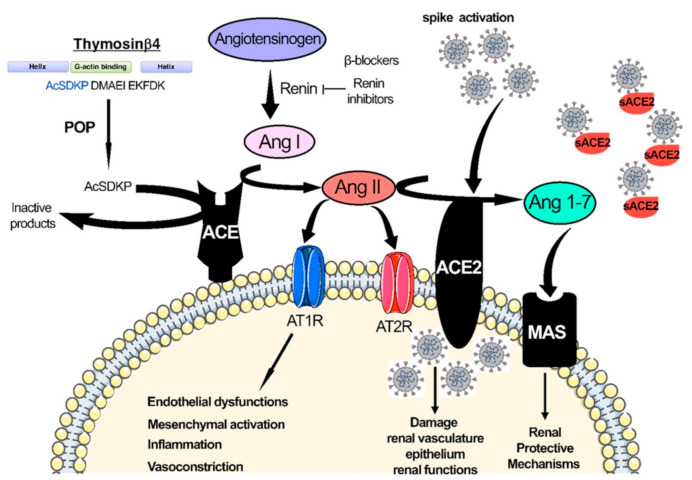
SARS-CoV-2 infection-associated disruption in the renin-angiotensin system.

Currently, there is no cure for diabetic glomerulosclerosis and diabetic nephropathy. The treatment for these conditions is lifelong, and these patients are at high risk of developing renal artery stenosis. Patients with DKD are often more anemic than patients with non-DKD. Inflammatory inhibitors of erythropoiesis and proteinuria have been implicated as contributing factors to this phenomenon [38]. In DKD, the interstitial compartment lesion may be an important factor that leads to increased production of erythropoietin in response to mounting hypoxia sensed by the kidney [38]. DKD patients, if suffering from inflammation, proteinuria, or both, become highly susceptible to chronic kidney disease (CKD)-associated with anemia. In diabetic patients, this tendency is further augmented, with a high prevalence of left ventricular hypertrophy (LVH) and heart failure [38]. In addition, insulin resistance in diabetic patients leads to a higher probability of kidney stone formation. This enhanced risk is also noted in obese and cardiometabolic syndrome patients [38]. Calcium-containing kidney stones are more frequent in diabetes, but the relative risk for uric acid nephrolithiasis is higher than in other stone types. Gluconeogenesis and glycogenolysis are impaired in patients with diabetes and kidney disease. These individuals are prone to both hypoglycemia and hyperglycemia. Persistent renin-angiotensin-aldosterone system (RAAS) activation and disruption in central metabolism encourage proteinuria and renal fibrosis [38,39,40]. There are recent reports of SARS-CoV-2 infections in diabetes and DKD [41,42,43,44,45,46,47,48]. Figure 1 demonstrates the role of RAAS in the understanding of SARS-CoV-2 in diabetes.

## 3. COVID-19 and Kidney Disease

Wu et al. conducted a study in 49 hospitalized patients on dialysis in Wuhan, China, and found that the disease course was generally more severe in patients with kidney failure than in those without kidney failure [18]. Hemodialysis patients received more noninvasive ventilation than non-dialysis patients and had a higher rate of complications, including acute respiratory distress syndrome (ARDS), shock, acute cardiac injury, and arrhythmia [49,50]. These findings suggest atypical clinical presentations of COVID-19 in patients on dialysis. The symptoms of fatigue and anorexia make the diagnosis difficult, since these symptoms are also observed in uremia, suggesting the need for a more systematic screening approach and universal respiratory precautions [49,50,51].

The authors described that 32% of kidney transplant patients (13/41) required hospitalization, had a significantly higher rate of dyspnea (77% vs. 21%; *p* = 0.003) and had substantially more elevated baseline creatinine (median of 2.0 vs. 1.3 mg/dL; *p* = 0.02) compared to nontransplant patients [49,50,51]. A study describes a practical approach to manage kidney transplants in COVID-19 patients [52].

A study suggests that the majority (96%) of hospitalized patients had a clinical course consistent with viral pneumonia, with 39% receiving mechanical ventilation, 21% requiring renal replacement therapy (RRT) with dialysis, while 28% of the kidney transplant recipients and 64% of intubated patients died [50].

As kidney transplant and dialysis patients with COVID-19 often present atypically, it is even more important to practice universal respiratory precautions [50,53]. Even after vaccines against SARS-CoV-2 are widespread, the global pandemic is likely to be our “new normal”, due to several more infectious variants [54,55,56].

## 4. COVID-19 Associated Nephropathy

**Figure 2:**
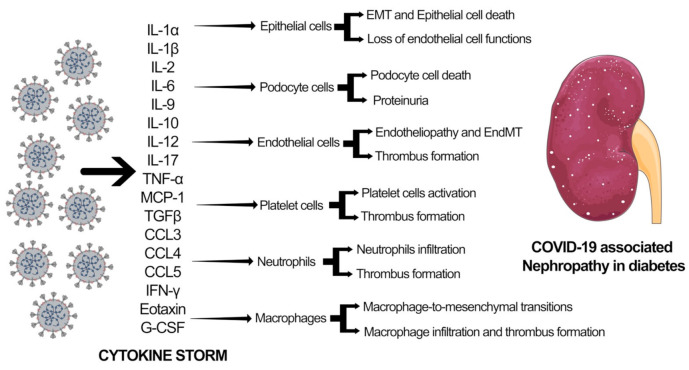
Mechanisms related to COVID-19 associated nephropathy in diabetes.

SARS-CoV-2 infection leads to downregulation of the ACE2 pathway resulting in myocardial injury, fibrosis, and inflammation. Several studies have linked SARS-CoV-2 infection with myocardial damage and heart failure, accompanied by ARDS, arrhythmias, coagulopathy, and acute kidney injury (AKI). It has been shown that SARS-CoV-2 affects the kidneys and causes podocyte injury that leads to proteinuria and release of proinflammatory cytokines and chemokines, including transforming-growth factor-β (TGF-β), Interferon gamma, interleukins, vascular endothelial growth factors (VEGF), platelet-derived growth factor (PDGF), and chemokine ligand 1 (CXCL1), that causes glomerular sclerosis, hyalinosis, mesangial matrix deposition, and fibrosis [57]. Increased glomerular filtration triggers oxidative stress that further exaggerates the glomerular injury through the deposition of the mesangial matrix and fibrosis. Glomerular nephrosis, AKI, and progressive chronic kidney disease (CKD) are the clinical signs of COVID-19-associated nephropathy with symptoms, such as fluid and electrolyte imbalance, hypertension, acid-base derangements, and edema [57]. Figure 2 delineates the effect of SARS-CoV-2 associated cytokine storm in different cell types in the kidney. Aberrations in cytokine and chemokine levels result in mesenchymal activations in epithelial cells, endothelial cells, and macrophages through epithelial-to-mesenchymal transition (EMT), endothelial-to-mesenchymal transition (EndMT), and macrophage-to-mesenchymal transition (MMT). These processes lead to podocyte cell death and severe proteinuria. Additionally, cytokine storm causes damage to endothelial cells, leading to endotheliopathy. EndMT-derived fibroblasts and endothelial cells interact with platelets and neutrophils and contribute to thrombus formation. This thrombus formation and accompanying excessive mesenchymal activation are crucial factors contributing to heart and kidney failure in diabetes.

## 5. MicroRNAs in Kidney Disease

MicroRNAs (miRNAs) are known for their crucial role in the kidney, and alteration in their expression level can lead to many pathological conditions, such as diabetes, DN, and CKD [58,59,60,61,62]. Therefore, miRNAs are promising therapeutic targets for kidney disease [37,58,59,63,64,65]. miRNAs also serve as potential markers for diagnosing and monitoring kidney disorders, such as DN and CKD [58,59,66,67].

MiR-30d, miR-140-3p, miR-532-3p, miR-194, miR-190, miR-204, miR-184, and miR-206 have been shown to be downregulated in progressive CKD [68,69]. Chung et al. demonstrated that miR-192 was highly upregulated in progressive kidney fibrosis [70]. miR-210 is a marker of renal allograft function [71]. Altered expressions of miR-223-3p and miR-93-5p have been observed in CKD [72,73]. The expression of urinary miR-196a is significantly higher in focal and segmental glomerulosclerosis (FSGS) [74]. There is a positive association between urinary miR-196a and proteinuria, glomerular filtration rate, renal fibrosis [74]. Patients with higher levels of urinary miR-196a have been shown to have a greater reduction in kidney function [74]. miR-29 regulates the expression of collagens and other genes related to the extracellular matrix, as well as profibrotic DPP-4 signaling and the interactions between DPP-4 and integrin β-1 [30,36,75,76,77,78,79]. The elevation of urinary miR-10a and miR-30d levels predicts they may be biomarkers for kidney disease [80]. miR-93 regulates VEGF expression in DKD pathogenesis [81]. miR-377 is linked with increased fibronectin expression [82]. miR-93 regulates TGF-β1-associated EMT and fibrogenesis [83]. miR-192 induces collagen synthesis in mesangial cells and promotes TGF-β-induced renal fibrosis in mice [70,84]. A meta-analysis revealed altered gene expression levels of microRNAs in renal fibrosis (five upregulated microRNAs include miR-142-3p, miR-21-5p, miR-223-3p, miR-214-3p, miR-142-5p, and two downregulated microRNAs miR-29c-3p and miR-200a-3p) [85]. microRNAs can exist in body fluids and function as endocrine signals to regulate the expression level of the target gene [86]. These microRNAs, in part, can present in extracellular vesicles, including exosomes [86,87]. In addition, urinary microRNAs derived from the kidney can passively filter through the glomerulus and be released from tubules into interstitial spaces [76,87,88,89,90]. The examples are miR-17, miR-451, miR-106a, and miR-19b establish themselves as biomarkers for diagnosis and prognosis in various diseases, including kidney diseases [90,91,92,93]. Hence, research on the microRNAs has gained momentum to establish themselves as biomarkers and offer the possibilities and perspectives for managing kidney diseases.

## 6. MicroRNAs in COVID-19 Disease

MicroRNAs are the key biomarker and have potential therapeutics against COVID-19 patients with or without diabetes. microRNAs can inhibit SARS-CoV-2 infection by targeting its protein-expressing genes related to structural and nonstructural proteins. microRNAs can inhibit viral DNA implication, suppression of cellular receptors, and inhibition of associated viral proteins [94]. However, less information is available about how microRNAs regulate SARS-CoV-2 gene expression in the host kidney cells to regulate viral DNA amplifications. Recent studies include the role of ID02510.3p-miRNA, ID00448.3pmiRNA, miRNA 3154, miRNA 7114-5p, miRNA 5197-3p, ID02750.3p-miRNA and ID01851.5p-miRNA, miR-5197-3p [95], miR-17-5p and miR-20b-5p [96] in the control COVID-19 pathogenesis by binding to the genome of SARS-CoV-2. Six miRNAs, including miR-21-3p, miR-195-5p, miR-16-5p, miR-3065-5p, miR-424-5p, and miR-421, were identified that can control all human coronaviruses by binding to the viral genome [97].

An elevated level of miR-1307-3p causes suppression in SARS-CoV-2 genome replication [98]. miR1307-3p regulates the level of antiapoptotic proteins like BCL2 and involves TGF-β signaling in chronic lung diseases [99].

Sardar et al. found six miRNAs targeting viral proteins: miR-let-7a and miR-101 target the nonstructural proteins, miR-126 and miR-378 target the N region, miR-23b targets the S region [100]. miR-29b-3p, miR-338-3p, miR-4661-3p, miR-4761-5p and miR-4793-5p can target S protein of SARS-CoV-2 [101]. Sardar et al. demonstrated the significance of cell-surface receptors, ACE2, in SARS-CoV-2 infection, and identified that miR-27b targets ACE2 receptors [100]. In another study, miR-200b-3p, miR-200 c-3p, and miR-429 were studied that can target ACE2. In addition, miR-98-5p and miR-let-7 clusters, as well as miR-4458, and miR-4500 also regulate the TMPRSS2 level. Diabetic patients are prone to SARS-CoV-2 infection because of elevated ACE2 receptor expression; therefore, blocking the ACE2 receptor can be used as a therapeutic target to combat COVID-19 [102].

In addition, microRNAs may improve immunity in COVID-19 patients. Therefore, search and identification of new endogenous microRNAs is required that may improve immunity and reduce the risk of SARS-CoV-2 infections.

## 7. Therapeutics and Perspectives

To date, coronavirus infection has been treated by a mixed medicine approach as there is no known single effective medicine against the virus. The antiviral drug-, remdesivir, has been used to treat SARS-CoV-2 infection [103,104,105,106]. However, RCTs for these drugs in diabetic and nondiabetic COVID-19 patients are urgently needed [103,105,106].

### 7.1. Targeting ACE2 Related Noncoding RNAs in COVID-19

**Figure 3:**
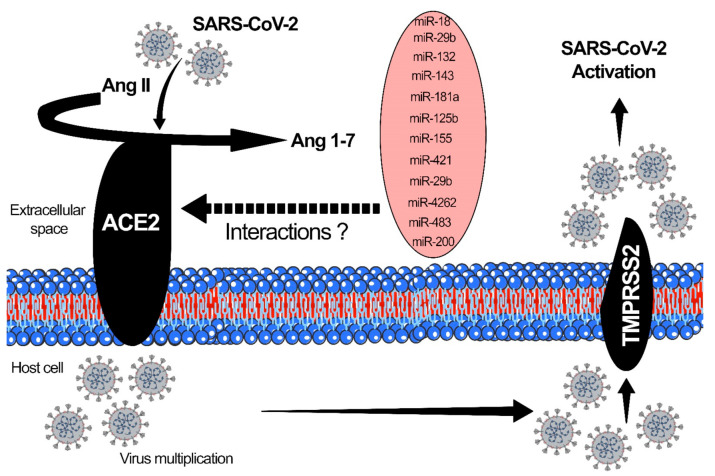
ACE2-related microRNAs.

The surface receptor ACE2 and TMPRSS2 participate in the endocytosis of viral particles into cells [53,57]. ACE2 receptors are highly expressed in the respiratory and gastrointestinal epithelium, heart, blood vessels, and kidneys. The SARS-CoV-2 virus enters into the host cell through these mechanisms: Adhesion, penetration, biosynthesis, maturation, and release of the virus [53]. The binding affinity of the virus to the host cells is correlated to the severity of the disease. After the binding, the host cell protease activates the S1 and S2 subunits of the S protein, and S2 protein facilitates the viral entry into host cells through endocytosis [57]. Once the virus enters the host cell and is uncoated, it undergoes nuclear replication, transcription, translation and is released from the cell after maturation [107]. ACE2 is a critical proinflammatory mediator in AKI and glomerular disorders associated with COVID-19 and is upregulated by miRNAs. ACE2 also plays a vital role in regulating oxidative stress in paraventricular nuclei. The rostral ventrolateral medulla serves as a lung receptor with a binding affinity for coronavirus, causing severe ARDS [57]. Altered ACE2 expression levels and activities have been implicated in several diseases, including hypertension, cardiovascular dysfunctions, and DKD [26]. DKD subjects show an elevated ACE/ACE2 ratio in the glomeruli and tubulo-interstitium, due to suppressed ACE2 levels [57]. Figure 3 demonstrates the interaction of SARS-CoV-2 with ACE2 and TMPRSS2. microRNAs, which have altered interactions with ACE2, are also shown in Figure 3.

MiRNAs that are known to alter the ACE2 expression level may be treated as potential targets to cure COVID-induced DKD. Some of the ACE2-associated microRNAs are as follows: miR-18a, which is expressed in mouse kidneys, targets ACE2 and is linked to hypoxia/reoxygenation in endothelial cell injury [57,108]. miR-125b is found in tubular epithelial cells, targets ACE2 and is linked to tubular apoptosis [109]. miR-143 is expressed in the kidney, heart, blood vessels, and lung, and is linked to AMP-activated protein kinase Kα2 via suppression of endothelial ACE expression through the phosphorylation of p53 and upregulation of miR-143/415 [57,110]. miR-145 is expressed in kidney, heart, and blood, regulates the kidney’s sympathetic nerve activity, and lowers renin levels by regulating ACE2 expression [111]. miR181a is expressed in human serum, and regulates the kidney’s sympathetic nerve activity, and lowers the renin level by regulating ACE2 expression [57,112]. miR-421 is expressed in heart, and is associated with remodeling and fibrosis by regulating ACE2 levels [113]. miR-4262 is expressed in lung tissue and is involved in acute lung injury through ACE2 regulation [110].

Long noncoding RNAs (LncRNAs) and microRNAs interactions influence disease phenotype in diabetic kidney disease [114]. A study by Radhakrishnan et al., 2020, has shown several lncRNAs to be differentially expressed in COVID-19 patient-derived lung tissue. LncRNA AC131011.2, AC007298.2, AC002398.2, AC022966.2, AC006064.4, AC099343.4, AC007032.1, AL034397.3, AC008537.4 were highly expressed, whereas some were suppressed includes LINC01089, LINC00115, AC027288.3, AC103706.1, AC022098.1, AC020915.3, AC007192.2, AP002840.2, AC018690.1, AC015819.1, AC009318.2, AC245140.2, AC097382.3, and AL035587.2 [115]. Some of the related observations were reported, such as higher expression level of metastasis-associated lung adenocarcinoma transcript 1 (MALAT1) and nuclear-enriched autosomal transcript 1 (NEAT1) lncRNAs in human tracheal epithelial cells after SARS-CoV-2 infections [115]. Moreover, Wei et al. studied the crucial role of MALAT1 in inflammation post-SARS-CoV-2 infections [116]. Knockdown of MALAT1 suppressed inflammation by lowering neutrophil chemotaxis and immune cell infiltration at the infection site. NEAT1 null mice displayed higher activation of NLRP3 and NLRC4 inflammasomes, which accelerated apoptosis. NEAT1 inhibition increased viral replication by inducing nucleus-to cytoplasm export of HIV-1 mRNA transcripts in HeLa cells [117]. All these studies indicate the role of lncRNAs in the progression of SARS-CoV-2 infection. Wen et al. have reported the role of circular RNA ‘circACTR2′ in inflammation and pyroptosis. circACTR2 inhibition decreased pyroptosis, interleukin (IL)-1*β* levels, collagen IV, and fibronectin production, indicating a role in the pathogenesis of DKD [118]. This study provides new insight into the pathogenesis of DKD and potential new therapeutic strategies. However, its pathogenic roles in DKD patients following COVID infections still need to be studied further.

### 7.2. Targeting Endothelial Dysfunction in COVID-19

Endothelial cells function to maintain vascular integrity, homeostasis, barrier function, and arrest inflammation by regulating interactions with immune cells and platelets [119]. Endotheliopathy, or endothelial dysfunction, is a key pathological characteristic in COVID-19 patients [120,121]. Both platelet and endothelial dysfunction are key components of COVID-19 pathology [120]. Autopsy specimens of blood vessels from COVID-19 patients have shown damage of endothelial cell and aberrant apoptosis [122,123]. It is not clear whether endothelial dysfunction is mainly a result of direct infection of endothelial cells by the SARS-CoV-2 virus or mediated through the indirect effects of cytokine storm during COVID-19 infection. However, biomarkers of endothelial damage, such as thrombomodulin, vWF, angiopoietin 2, and PAI1, are often elevated in COVID-19 patients, and have prognostic relevance [124,125]. Endotheliopathy leading to arteriopathy and thrombosis is a critical effector of the pathology of thrombotic complications observed in COVID-19 patients, including myocardial infarction and stroke [124,125].

Aging is one of the risk factors for COVID-19-associated death and is linked with endothelial dysfunction [126,127]. Oxidative and nitrosative-associated stress, which are elevated in older individuals, may accelerate endothelial dysfunction [128]. NADPH oxidases and mitochondria generate reactive oxygen species (ROS), and defective regulation of these pathways or loss of mitochondrial control generates ROS [127,129]. Aged endothelial cells display elevated ROS levels and decreased nitric oxide (NO) bioavailability, which is a well-known vasodilator promoter and has antiplatelets with cardiovascular protective properties [128,130]. Suppressed NO levels cause vasoconstriction and platelet activation and contribute to vascular diseases [131]. In addition, cerebromicrovascular dysfunctions are the least explored area in the context of COVID-19 pathology that arise, due to aging-associated endothelial dysfunction. Hyperactivation of poly(ADP-ribose) polymerase 1, which is usually seen after viral infections, leading to NAD^+^ depletion and consequently endothelial cell dysfunction [132]. In addition, aging-associated defects in oxidative stress resilience are caused by dysregulation of the nuclear factor erythroid 2-related factor 2 (NRF2) antioxidant defense pathways in endothelial cells, and this phenomenon may have a role in the disease process of COVID-19-related endotheliopathy [133]. Small molecules activators of NRF2 have been proposed as potential therapeutics for COVID-19 patients [134]. NRF2 dysfunctions increase endothelium damage effect of diabetes, conditions known to increase the risk of COVID-19-associated death [134]. The underlying mechanisms of aging-associated endothelial dysfunction involve multiple cellular pathways and are complex.

ACE2 expression is upregulated in response to interferons in human epithelial cells, which might contribute to potentiating the cellular uptake of SARS-CoV-2 [135]. However, single-cell analysis of human lungs has not reliably detected the levels of ACE2 expression in lung endothelial cells [135,136], which requires further investigation. Endothelial injuries, either by viral infections or by immune-mediated, are the critical features of severe COVID-19, and the underlying mechanisms require further investigations and in development of new therapeutics. Both the viral infection of endothelial cells by SARS-CoV-2 and the endothelial cell response to the inflammatory process caused with COVID-19 that induces immune cell response, aberrant cytokines production, and complement activation mediates endothelial cell damage and microvascular thrombosis [137].

Studies by a research group at Yale University led by Prof Hwa have demonstrated the role of endotheliopathy to COVID-19 severity, and suggested increased circulating levels of markers of endothelial cell damage, including thrombomodulin, angiopoietin 2, and vWF, are positively correlated with increased mortality in COVID-19 patients [138]. Several studies have also demonstrated the presence of acquired antiphospholipid antibodies in COVID-19 patients [139]. Antiphospholipid antibodies, such as lupus anticoagulant, anticardiolipin antibody, and anti-β2-glycoprotein I, predispose individuals to be arterial and venous thrombosis [139]. The cases studies reported multiple cerebral infarctions in the setting of positive antiphospholipid antibodies [140]. In another study, the percentage of patients testing positive for a lupus anticoagulant was significantly higher among patients with COVID-19 when compared to individuals who have no COVID-19 [141]. This evidence indicates that antiphospholipid antibodies interact with multiple vascular and haematopoietic components, including endothelial cells, platelets, and complement factors, to accelerate thrombosis [142,143]. Although antiphospholipid antibodies are a common occurrence in viral infections, and are often transient and do not always imply a higher risk of thrombosis [144]. Moreover, false-positive results in lupus anticoagulant tests can arise in patients receiving anticoagulation therapeutics [145]. However, antiphospholipid antibodies play a crucial factor in the pathophysiology of thrombosis-linked with COVID-19 requires further research.

In our laboratory, we have investigated crucial molecules that provide endothelial cell stability integrity, such as endothelial SIRT3, which is an essential mitochondrial protein and maintains the balance of fuel preference between healthy and damaged endothelial cells in diabetes [39,146,147]. Another study demonstrates that endothelial FGFR1 signaling is also important for endothelial cell heath in diabetic kidneys and the heart [148,149]. We have demonstrated the significance of the glucocorticoid receptor (GR) in endothelial cell health, endothelial cell stability, endothelial cell permeability, and leakage [150,151]. The GR agonist dexamethasone is given to SARS-CoV-2 patients to suppress inflammation; however, this drug can have severe adverse effects in diabetic patients. Therefore, the use of nonsteroidal GR activators is preferred to protect against thrombus formation, suppress inflammation and mitigate mesenchymal activation [150,152]. In addition, nonsteroidal GR activators could help restore endothelial stability and neighboring cell homeostasis and could be useful as a treatment option [150]. An endogenous peptide, N-acetyl-seryl-aspartyl-proline (AcSDKP), has shown promise in the preclinical setting by providing benefit to diabetic endothelium and other cell types [21,22,79,148,153]. However, further studies are required to confirm the therapeutic efficacy of AcSDKP in diabetic COVID-19 endothelium. In addition, podocyte-endothelial crosstalk regulated by glucocorticoid receptors is important for the glomerular health therefore, the search for the new safe non-steroidal GR agonists is needed for development of future generation medication against diabetic nephropathy [154] and such medications could have beneficial effects to COVID-19 patients. Moreover, any medication that supports endothelial cell heath, may be of benefit to diabetic COVID-19 patients.

### 7.3. Targeting Dipeptidyl Transferase-4 in COVID-19

There is limited data available on the potential benefits or risks of insulin or antidiabetic agents in patients with acute SARS-CoV-2 infection [155]. GLP1-RA have been proven safe and effective for blood glucose control [155]. GLP1-RA attenuate pulmonary inflammation, reduce cytokine over-production, and reduce lung injury [129]. However, intensive research needs to be conducted on the use and safety of these agents for critically ill patients or patients with SARS-CoV-2 infection [155].

**Figure 4:**
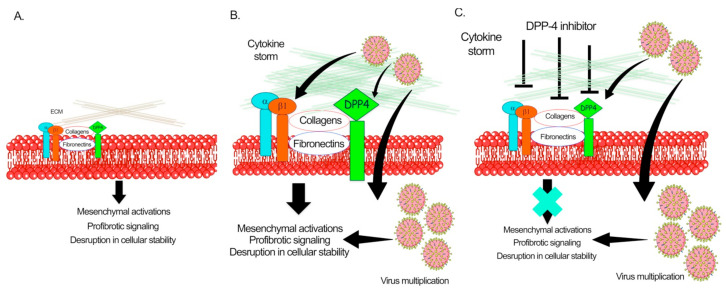
Pathological role of dipeptidyl transferase-4 in COVID-19 associated nephropathy in diabetes. (**A**) Healthy kidney cells. (**B**) SAR-CoV-2 infection and associated cytokine storm elevates DPP-4-Integrin-β1 interactions, induce mesenchymal activations and profibrotic signaling in the kidneys cells subsequently lead to higher ECM deposition, collagens and fibronectin accumulation. (**C**) DPP-4 inhibition cancels the mesenchymal transition processes and, suppresses pro-fibrotic signaling, ECM deposition, fibronectin and collagen accumulation. Servier medical art illustrations were used to design the figures.

Solerte et al. has shown that, in diabetic patients who have COVID-19 disease, treatment with sitagliptin is linked to reduced mortality and better clinical outcomes [156]. Sitagliptin is an oral dipeptidyl peptidase 4 (DPP-4) inhibitor, used to treat type II diabetes, and studies have suggested that the SARS-CoV-2 virus may interact with DPP-4 when entering cells [156]. Analysis of the structure, receptor binding, and receptor modeling of the SARS-CoV-2 virus, it has also been postulated that DPP-4 can facilitate the virus entry into target cells because of its high homology with Middle-East respiratory syndrome coronavirus [156]. In the study conducted by Solerte et al. on 338 patients, sitagliptin (DPP-4 inhibitor) treatment was analyzed and compared with untreated control subjects [156]. All subjects had pneumonia and oxygen saturation <95%. As standard care, patients were given metformin, but also glibenclamide, DPP-4 inhibitors, sodium-glucose cotransporter 2 inhibitors, GLP-1 receptor agonists, glinides, and thiazolidinediones [156]. Overall, 169 patients received DPP-4 inhibitor (sitagliptin) and standard care, whereas 169 controls were given only standard care [156]. Results demonstrated that diabetic patients with sitagliptin treatment had reduced mortality, improvement in clinical outcomes, and have lower blood glucose levels, when compared to diabetic patients receiving only standard care, [156]. Linagliptin, which was observed to more effective in preclinical settings of DKD [27,78], should be evaluated further for RCTs in COVID-19 diabetic and nondiabetic subjects. Figure 4 depicts the pathogenic role of DPP-4 in the pathogenesis of SARS-CoV-2. However, further studies are required to establish the critical role of DPP-4 in viral infections.

### 7.4. Targeting AMP-Activated Protein Kinase in COVID-19

The SARS-CoV-2 virus enters the human body through interactions with its spike protein (S1) and the N-terminal region of ACE2 [157]. The receptor-binding domain (RBD) of the virus binds with the protease domain (PD) of the ACE2 receptor and forms an RBD-PD complex [157]. Animal studies suggest the implication of ACE2 in the SARS-CoV-2 associated acute lung injury (ALI) [157]. AMPK elevates the expression level of ACE2 and promotes its stability by phosphorylation. AMPK activation is one of the mechanisms combating diabetes and associated complications [158]. Metformin-associated AMPK activation leads ACE2 phosphorylation [157]. ACE2 phosphorylation suppresses the binding with the SARS-CoV-2 RBD, due to steric hindrance [157]. Moreover, virus infection itself causes suppression in ACE2 receptors expression. This, in turn, causes an imbalance in RAS, accelerating the inflammatory and profibrotic processes, promoting lethal cardio-pulmonary complications [157]. By upregulating ACE2, the imbalance in RAAS could be abolished. Therefore, metformin would help not only in preventing SARS-CoV-2 entry, but also in preventing detrimental sequelae by causing activation of ACE2 through AMPK-signaling [157]. Moreover, known effective antidiabetic agents related to AMPK pathways should be analyzed for their beneficial effects in diabetic COVID-19 patients [159,160,161,162]. Further preclinical, small- and large-scale RCTs are needed to analyze the use of AMPK activators in diabetic and nondiabetic SARS-CoV-2 patients.

## 8. Conclusions

This review described the direct and indirect mechanisms of COVID-19 severity in diabetes and DKD. SARS-CoV-2 infections cause strong cytokine storms, which either directly or indirectly disrupt the organ-protective mechanisms in diabetic patients. Targeting endotheliopathy, pathogenic DPP-4, AMPK activators, and ACE2-related noncoding RNAs has shown promise in some studies in the treatment of DKD patients [163]. However, further studies are needed to determine whether these small molecules modulators can be further used as a supplement to the standard care of COVID-19. These results will identify essential regulators and their biological functions, which are important for the stability of whole organs and specific cell types in diabetes.

## Data Availability

Data sharing not applicable.

## Abbreviations

AcSDKP	*N*-acetyl-seryl-lysyl-proline
ACE	Angiotensin-converting enzyme
ACE2	Angiotensin-converting enzyme 2
AMPK	AMP-activated protein kinase
ARDS	Acute respiratory distress syndrome
ALI	Acute lung injury
CKD	Chronic kidney disease
COVID-19	Coronavirus disease 2019
CXCL1	Chemokine ligand 1
DPP-4	Dipeptidyl transferase-4
DKD	Diabetic kidney disease
DM	Diabetes mellitus
DN	Diabetic nephropathy
ECM	Extracellular matrix
EMT	Epithelial-to-mesenchymal transition
EndMT	Endothelial-to-mesenchymal transition
ESRD	End-stage renal disease
FGFR1	Fibroblast growth factor receptor 1
GLP-1	Glucagon-like peptide-1
GR	Glucocorticoid receptor
KRT	Kidney replacement therapy
LncRNA	Long noncoding RNAs
LNA	Locked nucleic acid
MALAT1	Metastasis-associated lung adenocarcinoma transcript 1
MiRNA	MicroRNA
MMT	Macrophage-to-mesenchymal-transition
NEAT1	Nuclear enriched abundant Transcript-1
NLRP3	NOD-, LRR- and pyrin domain-containing protein 3
PDGF	Platelet-derived growth factor
RAAS	Renin angiotensin system
RBD	Receptor binding domain
RCT	Random clinical trials
SIRT3	Sirtuin 3
SARS-CoV-2	Severe acute respiratory syndrome coronavirus 2
TMPRSS2	Transmembrane protease serine 2 receptors
TGFβ1	Transforming growth factor β1
TNF α	Tumor necrosis factor α
VEGF	Vascular endothelial growth factor
